# Prosthodontics Awareness Among Medical Faculty: A Multicentric Cross-Sectional Study in North and Eastern India

**DOI:** 10.7759/cureus.104239

**Published:** 2026-02-25

**Authors:** Surender Kumar, Bishnupati Singh, Madhu Ranjan, Amit V Mahuli, Irfanul Huda, Sanjay Kumar

**Affiliations:** 1 Department of Prosthodontics, Dental Institute, Rajendra Institute of Medical Sciences, Ranchi, IND; 2 Department of Public Health Dentistry, Dental Institute, Rajendra Institute of Medical Sciences, Ranchi, IND; 3 Department of Prosthodontics, Patna Dental College and Hospital, Patna, IND; 4 Department of Prosthodontics, Lady Hardinge Medical College, New Delhi, IND

**Keywords:** awareness, health care professional, medical teaching faculty, prosthodontic knowledge, survey and questionnaire

## Abstract

Introduction: Prosthodontics, a specialized branch of dentistry, plays a crucial role in oral health and overall well-being. However, awareness of its scope and importance among medical professionals outside the dental field remains understudied. Thus, the aim was to assess and compare the knowledge and awareness of medical faculty regarding prosthodontics as a dental specialty across three postgraduate medical colleges in India.

Methodology: A cross-sectional questionnaire survey was conducted among medical teaching faculty at three postgraduate medical colleges in India. A self-constructed, expert-validated questionnaire comprising 16 close-ended questions assessed knowledge and awareness of prosthodontics. A pilot study was performed to validate the questionnaire and identify potential implementation issues. Data collected from the three medical colleges were subjected to statistical analysis.

Results: A total of 125 medical faculty members participated in the study, with a mean age of 41.2 ± 8.88 years and an average teaching experience of 10.2 ± 7.43 years. Knowledge regarding prosthodontics was notably high, with 102 (81.6%) correctly identifying its role in managing missing teeth and 116 (92.8%) recognizing subdivisions within the specialty. Implant-supported prostheses (n=59, 47.2%) and crowns and bridges (n=35, 28%) were the most preferred replacement methods. Referrals predominantly targeted prosthodontists for missing teeth (n= 90, 72%), crowns (n=104, 83.2%), and surgical reconstruction of oral structures (n=85, 68%), whereas plastic surgeons were primarily referred to for extra-oral prostheses (n= 78, 62.4%). Institutional differences significantly influenced referral patterns, particularly for missing teeth to prosthodontists (p = 0.028) and extra-oral prosthesis to plastic surgeons (p = <0.001).

Conclusion: While the majority of faculty members possess knowledge and awareness of the field of prosthodontics, it is imperative to address certain key areas of prosthodontists' work to the teaching faculty of medical colleges across India.

## Introduction

Oral health, despite being a critical component of systemic health, is frequently overlooked [1,2]. Research indicates that medical practitioners and nurses play a vital role in an individual's oral healthcare, and a lack of knowledge and awareness can lead to the deterioration of the patient's condition [3,4]. A significant portion of the population consults general medical practitioners for oral health issues, who are expected to refer patients to appropriate dental specialists for management. Therefore, it is imperative that they possess a comprehensive understanding of the various dental specialties. Although the medical and dental professions share a common biomedical foundation, evidence suggests that there are substantial deficiencies in basic oral health knowledge and related disciplines among medical practitioners [5]. The gap in the integration of medical and dental care in our country is apparent and necessitates the attention of both fields [6,7]. This gap indirectly impacts treatments that require interdisciplinary planning and approaches. Dental and medical practitioners are essential components of a comprehensive healthcare team; however, the existence of such collaboration in India remains questionable. Dentistry encompasses various specialties, including oral and maxillofacial surgery, endodontics, periodontics, prosthodontics, orthodontics, paediatric dentistry, oral and maxillofacial pathology, oral medicine and radiology, and public health dentistry [8].

Prosthodontics is a dental specialty focused on the diagnosis, treatment planning, rehabilitation, and maintenance of oral function, comfort, appearance, and health in patients with clinical conditions related to missing or deficient teeth and/or maxillofacial tissues, utilizing biocompatible substitutes [9]. The loss of multiple teeth often compromises facial aesthetics, speech, mastication, and the psychological well-being of individuals. Various treatment options are available, including removable or fixed partial dentures, implants, and complete dentures or overdentures. Therefore, the careful selection and design of an appropriate dental prosthesis are essential to rehabilitate the condition and achieve a normal, healthy quality of life [10].

Prosthodontics has evolved to encompass tooth replacement through implants and computer-aided design and computer-aided manufacturing (CAD/CAM) technology. The introduction of new methodologies and the advent of digitalization have rendered traditional procedures, such as impression taking and model pouring, less central to prosthesis fabrication. Despite these advancements, the perception of Prosthodontics as a specialty among medical professionals remains unclear. Enhancing awareness of Prosthodontics within the medical community is crucial, as it would significantly benefit the scope and rehabilitation of patients requiring tooth replacement and other specialized services related to Prosthodontics

There is a notable scarcity of published literature concerning the knowledge and information related to prosthodontics among medical faculty [11]. Therefore, the present study aims to assess and compare the knowledge and awareness of prosthodontics as a dental specialty among the medical faculties of three postgraduate medical colleges in India.

## Materials and methods

Study design and setting

A multi-centre cross-sectional survey utilizing a questionnaire was conducted among registered medical professionals employed at three government postgraduate medical colleges (PGMC-I, PGMC-II, and PGMC-III) situated in the eastern and northern regions of India. The study was carried out from March 2022 to July 2022, employing convenience sampling. The methodology adhered to the STROBE reporting guidelines. Ethical approval for this study was obtained from the Institutional Ethical Committee of PGMC-I (IEC401/29/12/2021).

Study participants

The selection criteria encompassed registered medical doctors, both male and female, who are faculty members aged between 25 and 72 years and are employed at the selected institutions.

Data sources/measurement

Following a comprehensive literature review, a meticulously structured questionnaire was developed through consultations with medical faculty experts. Both face validity and content validity were evaluated by a panel of six experts, leading to the creation of a prototype questionnaire. This prototype underwent pretesting with 10 faculty members from the PGMC-I to assess feasibility. Subsequent modifications were made, reducing the questionnaire to 16 items. The initial three questions evaluated knowledge pertaining to the speciality, questions four through nine assessed awareness, and questions ten to sixteen focused on practice patterns. After a two-week interval, the questionnaire was re-administered to the same faculty members to determine test-retest reliability (r=0.82). The self-administered questionnaire consisted of 16 closed-ended questions, including demographic information such as age, designation, specialty, and institutional affiliation. The questionnaire was distributed to 165 medical doctors across three institutions in the northern, northeastern and eastern parts of India having close affinity to dental institutions. The study details were thoroughly explained to each participant, assuring them of the confidentiality and anonymity of their responses. Informed consent was obtained from all participants.

Inclusion Criteria

All medical doctors and faculty members who work at the chosen institutions are currently practicing. Participants give their informed consent willingly after being told about the study's goals and how their privacy will be protected. Employees who were at the institution during the time that data was collected.

Exclusion Criteria

Administrative staff, lab technicians, or healthcare workers who do not have a medical degree (MBBS or higher). The 10 PGMC-I faculty members who took part in the pretesting and reliability (test-retest) phase to avoid bias or the "recall effect". Participants who send back the questionnaire with more than a certain percentage (like 20%) of questions unanswered or missing demographic information.

Bias

Participants were required to complete the questionnaires under the supervision of an investigator. The investigators at each centre were tasked with the distribution and collection of the completed questionnaires.

Study size estimation

 Based on a pilot study with an alpha error of 5% and a power of 80% as reported in previous literature, and employing a 95% confidence interval using the formula N= (Z2 × P (1 × P))/e2, the sample size was calculated to be 55 per centre. Consequently, a total of 165 participants were included in this study.

Statistical analysis

The data were transferred to Microsoft Excel and subsequently analyzed using the statistical package Jamovi (Version 2.5) R-based, user-friendly, open-source platform. Descriptive statistics were employed to calculate frequency distributions, and the chi-square test was utilized to assess relationships among variables, with statistical significance determined at p < 0.05.

## Results

Participant demographics

Out of 165 distributed questionnaires, 125 were completed by medical faculty members, yielding a response rate of 75%. The participants had a mean age of 41.2 ± 8.88 years, with an age range of 26 to 61 years, and an average teaching experience of 10.2 ± 7.43 years, ranging from 2 to 35 years. The sample consisted of 66.4% males (n=83) and 33.6% females (n=42). The distribution across institutions was relatively balanced: 35.2% from PGMC I (n=44), 36% from PGMC II (n=45), and 28.8% from PGMC III (n=36), presented in Table 1. Response questions are presented in Table 2 as survey results in percentages from question 1 to question 16.

**Table 1:** Participant demographic characteristics. The demographic variables as age, teaching experience, gender, and postgraduate medical college (PGMC) in all participants (N=125).

Characteristic	Mean ± SD (Range) / n (%)
Age (years)	41.2 ± 8.88 (26-61)
Teaching Experience	10.2 ± 7.43 (2-35)
Male	83 (66.4%)
Female	42 (33.6%)
PGMC I	44 (35.2%)
PGMC II	45 (36.0%)
PGMC III	36 (28.8%)

Knowledge regarding prosthodontics

In the study, 81.6% (n=102) of participants accurately identified prosthodontics as the appropriate field for addressing missing teeth. The level of knowledge regarding prosthodontics and its subdivisions was notably high, with 92.8% (n=116) of respondents recognizing specific areas such as implant tooth replacement, fixed tooth replacement, and maxillofacial prostheses. Furthermore, all respondents (100%, n=125) correctly identified the consequences associated with missing teeth (Table 2).

In terms of preferred methods for tooth replacement, 47.2% (n=59) of participants favoured implant-supported prostheses, while 28% (n=35) preferred crowns and bridges. Additionally, approximately half of the participants (50.4%, n=63) accurately identified prosthodontics as the specialty associated with dental laminates and veneers (Table 2).

**Table 2:** Frequency distribution of responses assessing awareness, knowledge, and referral practices related to Prosthodontics. The responses from questions 1-16 by all study participants (N=125).

S.No.	Question	Response Category	(n)	(%)
1	Dental specialty “Prosthodontics” deals with:	Carious teeth	9	7.2
		Mal-aligned teeth	14	11.2
		Missing teeth	102	81.6
2	Does Prosthodontics as a sub-specialty have subdivisions?	Yes	116	92.8
		No	9	7.2
2a	If yes, which Prosthodontic subdivisions do you know about?	All subdivisions	107	85.6
		Fixed prosthesis	2	1.6
		Removable prosthesis	2	1.6
		Implant tooth replacement	3	2.4
		Maxillofacial prosthesis	2	1.6
		Not applicable	9	7.2
3	What are the consequences of missing or affected teeth?	All consequences	125	100.0
4	Preferred mode of replacement of missing teeth	Crown and bridge	35	28.0
		Implant-supported prosthesis	59	47.2
		Removable denture	6	4.8
		Not sure	25	20.0
5	Dental laminates and veneers are indicated for tooth repair in which specialty?	Prosthodontics	63	50.4
		Conservative & Endodontics	17	13.6
		Orthodontics	7	5.6
		Not sure	38	30.4
6	Which dental specialty deals with implants?	Prosthodontics	62	49.6
		Oral & Maxillofacial Surgery	5	4.0
		Periodontics	6	4.8
		Orthopaedics	1	0.8
		All	51	40.8
7	Do you think oral hygiene maintenance is required for prosthesis?	Yes	123	98.4
		No	2	1.6
8	Can medical/systemic disorders lead to loss of teeth?	All	109	87.2
		Osteoporosis	8	6.4
		Osteoporosis (others)	6	4.8
		Diabetes mellitus with CVA	2	1.6
9	Replacement of missing teeth is a:	Multiple sittings procedure	78	62.4
		Two sittings procedure	9	7.2
		All	38	30.4
10	Patients with missing teeth should be:	Referred to Prosthodontist	90	72.0
		Referred to Dental Institute	19	15.2
		Referred to General Dentist	16	12.8
11	Patients demanding crown placement should be:	Referred to Prosthodontics	104	83.2
		Referred to Orthodontics	10	8.0
		Referred to Endodontics	8	6.4
		Referred to Oral Surgery	3	2.4
12	Patients with surgical resection of palate/mandible should be:	Referred to Prosthodontics	85	68.0
		Referred to Plastic Surgeon	33	26.4
		Referred to Cosmetologist	2	1.6
		None	5	4.0
13	Is oral examination a part of your routine examination?	Yes	87	69.6
		No	38	30.4
14	Do you think replacement of missing teeth is essential?	Definitely yes	92	73.6
		Difficulty in mastication	26	20.8
		No	7	5.6
15	For eye, ear, finger, and facial prosthesis, patients should be:	Referred to Plastic Surgeon	78	62.4
		Referred to Prosthodontics	38	30.4
		Don’t know	6	4.8
		Referred to ENT	3	2.4
16	Whom have you or your family visited for replacement of missing teeth?	General Dentist	63	50.4
		Prosthodontist	53	42.4
		Orthodontist	8	6.4
		Endodontist	1	0.8

Awareness

In response to inquiries regarding specialties involved in dental implant procedures, 49.6% (n=62) of respondents specifically identified prosthodontics, whereas 40.8% (n=51) believed that all dental specialties are involved in managing implants. A significant majority (98.4%, n=123) underscored the critical importance of maintaining oral hygiene for dental prostheses. Additionally, most participants (87.2%, n=109) demonstrated awareness of the systemic diseases associated with tooth loss. In this study, 62.4% (n=78) of participants perceived the process of replacing missing teeth as necessitating multiple appointments. In a routine medical assessment, oral examinations were conducted by 69.6% (n=87) of the respondents. Concerning the importance of replacing missing teeth, a significant majority (73.6%, n=92) strongly endorsed the necessity, primarily to prevent difficulties in mastication. (Table 2)

Referral patterns

Furthermore, 72% (n=90) of respondents referred patients with missing teeth specifically to prosthodontics. Additionally, 83.2% (n=104) referred patients requiring crowns to prosthodontists. Patients in need of surgical reconstruction of the palate or mandible were predominantly referred to prosthodontists (68%, n=85), followed by plastic surgeons (26.4%, n=33) In the context of extra-oral prostheses, such as those for eyes, ears, fingers, and faces, 62.4% (n=78) of patients were referred to plastic surgeons, while 30.4% (n=38) were directed to prosthodontists as the appropriate specialists (Table 2).

Significant statistical differences were identified in patient referral patterns across institutions, particularly in relation to referrals for missing teeth (χ² = 10.8, p = 0.028). In contrast, other referral patterns, despite their variability, did not exhibit statistically significant differences among the institutions (p <0.05), as presented in Table 3. The findings demonstrate a comprehensive understanding and awareness of prosthodontics among the medical faculty, underscoring specific areas that warrant further targeted educational interventions.

**Table 3:** Referral patterns by institution. (*p < 0.05 significant. The table depicts only the highest observed frequencies for each of the questions pertaining to referral.

Referral Context	PGMC I n (%)	PGMC II n (%)	PGMC III n (%)	Chi square value	Degree of freedom	p-value
Missing teeth (Prosthodontics referral)	24 (54.5%)	36 (80%)	30 (83.3%)	10.8	4	0.028*
Crown placement (Prosthodontist)	39 (88.6%)	35 (77.8%)	30 (83.3%)	9.64	6	0.141
Extra-oral prosthesis (Plastic Surgeon)	16(20.5%)	32(41.4%)	30(38.5%)	29.6	6	<0.001*
Surgical Reconstruction (Prosthodontist)	30 (35.3%)	31 (36.5%)	24 (28.2%)	1.50	6	0.960

## Discussion

The competencies and scope of prosthodontists are not well comprehended by the general public and medical practitioners. There is minimal awareness among practitioners regarding this treatment modality, which is encompassed within the curriculum of specialized dentistry. Consequently, due to this lack of awareness, patients with missing teeth, associated oral structures, and maxillofacial defects are not adequately directed and referred to a prosthodontist for rehabilitation. Therefore, the present study was conducted to gather data concerning the awareness and knowledge of medical practitioners, particularly those in medical colleges, regarding the maxillofacial branch of dentistry as a specialty for restoring maxillofacial defects and addressing other related issues.

A significant proportion of respondents (81.6%) demonstrated awareness that the branch primarily focuses on the replacement of missing teeth and recognized that this process typically involves multiple visits. Furthermore, a majority (92%) were knowledgeable about the subdivisions of prosthodontics and the various modalities for artificial tooth replacement. In a previous study conducted in Chennai among medical practitioners, 279 participants (93%) correctly identified Prosthodontics as a dental specialty [12]. However, approximately 61 participants (41.5%) were unaware of the appropriate timing for routine dental consultations [10].

Implant-supported prostheses are generally regarded as the preferred method for tooth replacement. However, there remains some uncertainty, as evidenced by 20% of respondents expressing ambiguity regarding their preferred mode of tooth replacement. Enhancing public awareness of dental implants could facilitate more informed decision-making concerning oral health. In a particular study, 70.9% of participants expressed interest in learning about dental implants; nonetheless, awareness levels differ across countries, with some studies indicating low awareness among participants [9]. Implant treatment is primarily associated with prosthodontics, with only 50% of respondents concurring with this association, while 48% linked it to other specialties such as Oral and Maxillofacial Surgery (OMFS), Periodontics, and Orthodontics. The medical community typically refers patients requiring tooth and crown replacement to prosthodontists for rehabilitation.

The majority of respondents were unable to associate laminates and veneers with the field of prosthodontics. The study indicated that although dental practitioners are acquainted with laminate veneers, they lack awareness of recent advancements and potential causes of failure.

This underscores the necessity for enhanced education to improve their efficacy and address practical challenges [13]. Prosthetic rehabilitation following surgical resection is still predominantly considered the domain of plastic surgeons rather than prosthodontists. General dentists are more frequently sought by specialty dentists for dental treatment for themselves and their family members of medical practitioners. This suggests a general lack of awareness among medical faculty regarding the clinical scope and contributions of prosthodontists. Many medical doctors hold the belief that all dental-related issues can be addressed by general dental practitioners. It is imperative to increase awareness among medical trainees and doctors about the various subspecialties of dentistry to ensure appropriate referral practices for dental patients they encounter [8].

Analysis of the study's findings reveals a notable deficiency in both knowledge and confidence regarding the management of dental diseases. The responses from the studies indicate a discernible gap in essential knowledge related to the assessment and management of prosthodontic treatment among medical doctors. From the perspective of medical education, the foundational knowledge acquired through basic dental education is crucial for medical doctors studying prosthodontics [5].

As a specialized branch, the divisions play a crucial role in raising awareness about the various treatment options available for the replacement of missing teeth. The increasing preference for dental implants as a mode of tooth replacement is likely to enhance the acceptance of this treatment within the field of prosthodontics. Since the introduction of implants for addressing tooth loss, prosthodontics has been widely recognized as the specialty responsible for implant management, which is a positive development and a source of encouragement. It is imperative to identify systemic diseases that lead to tooth loss and prioritize referrals to prosthodontists for early and effective management. Prosthodontists should be acknowledged by medical healthcare professionals as specialists in the replacement of missing teeth [10,11].

It is imperative that oral examinations be mandated for all patients visiting medical practitioners, akin to the standard practice of conducting medical examinations in routine prosthodontic procedures. To enhance the understanding and recognition of prosthodontic specialties among healthcare professionals, it is recommended that awareness campaigns and dental education programs be organized in collaboration with medical colleges and the Indian Medical Association (IMA). While the removal of oral cancer, cysts, and tumors by surgeons specializing in medical fields is common, coordination with prosthodontists is essential. Such collaboration will enhance rehabilitation with prostheses by elucidating the role of teeth and soft tissues in supporting prosthetic devices for optimal functionality [14].

The psychosomatic significance of teeth and associated oral structures in the overall well-being of the body should be comprehended by medical practitioners, thereby ensuring their preservation and optimal replacement. If the medical education curriculum incorporated more comprehensive information and skill development in Prosthodontics, medical doctors would gain a better understanding of the importance of early intervention, potentially leading to more favorable outcomes for patients requiring dental prosthetics [15-17].

A cross-sectional study revealed that medical practitioners possess substantial knowledge regarding dentistry, with 76.3% recommending that their patients visit a dentist biannually [12]. However, the response rate may compromise the study's statistical power.

Consequently, the findings of this study should not be deemed definitive. The absence of data on non-respondents raises the possibility of response bias, as participants may predominantly include those favorably inclined towards the study's objectives. Therefore, these results should be interpreted with caution.

This study represents the first investigation among medical practitioners concerning their knowledge, attitudes, and practices related to the prosthodontics dental specialty. Consequently, there are no available comparisons with similar studies. Nonetheless, research has been conducted on general practitioners' knowledge regarding various oral diseases. Routine oral examinations and referrals for tooth replacement in patients are likely to encourage them to pursue treatment.

Recommendation

Enhancing awareness of prosthodontics among medical faculty necessitates a comprehensive approach. Faculty sensitization programs can be implemented through interdisciplinary workshops and Continuing Medical Education (CME) sessions to enhance understanding of prosthodontic treatment modalities. Incorporating fundamental dental and prosthodontic education into undergraduate medical curricula would facilitate early interdisciplinary exposure. Promoting collaborative case management and joint discussions between medical and dental departments, particularly in teaching hospitals, can foster improved interdisciplinary cooperation. At the policy level, medical education regulatory bodies, such as the National Medical Commission (NMC), should consider integrating structured orientation modules on dental specialties, particularly prosthodontics, during faculty induction. To substantiate these findings and inform national-level curriculum reforms, further multicenter studies with larger and more diverse samples are recommended. These initiatives can help bridge the knowledge gap and enhance interdisciplinary patient care.

Limitations

This study offers valuable insights; however, it is constrained by specific limitations that necessitate a prudent interpretation of the results. The study was limited to three postgraduate medical colleges in Northern and Eastern India, thereby constraining the applicability of the findings to the national context, where educational exposure and interdisciplinary practices may vary. The use of convenience sampling and a small sample size (n=125) raises the possibility of selection bias, as faculty members with a prior interest in dentistry may have been more likely to take part. Additionally, the cross-sectional design provides merely a temporal snapshot, hindering the evaluation of causal relationships, and the utilisation of self-administered questionnaires renders the data vulnerable to recall and social desirability biases.

## Conclusions

The study reveals significant gaps in knowledge, diagnostic abilities, and confidence among medical faculty regarding oral diseases and prosthodontic treatments across three postgraduate medical institutes in India. While there is a general inclination to refer patients to prosthodontists for specific treatments, inconsistencies in awareness and referral practices were observed across departments and designations. Clinical faculty demonstrated relatively better understanding compared to paraclinical and preclinical counterparts, highlighting the importance of practical experience in enhancing interdisciplinary knowledge. The findings underscore the need for improved interdisciplinary awareness and collaboration between medical and dental professionals, particularly in the field of prosthodontics.
